# Solar-powered oxygen delivery for the treatment of children with hypoxemia: protocol for a cluster-randomized stepped-wedge controlled trial in Uganda

**DOI:** 10.1186/s13063-019-3752-2

**Published:** 2019-12-05

**Authors:** Nicholas Conradi, Qaasim Mian, Sophie Namasopo, Andrea L. Conroy, Laura L. Hermann, Charles Olaro, Jackson Amone, Robert O. Opoka, Michael T. Hawkes

**Affiliations:** 1 Department of Pediatrics, University of Alberta, 3-588D Edmonton Clinic Health Academy, 11405 87 Ave NW, Edmonton, Alberta T6G 1C9 Canada; 2 Kabale Regional Referral Hospital, Kabale, Uganda; 3 Indiana University, Indianapolis, USA; 4 Kelowna General Hospital, Kelowna, Canada; 5 Ministry of Health, Kampala, Uganda; 6 Department of Paediatrics and Child Health, Mulago Hospital and Makerere University, Kampala, Uganda; 7 Department of Medical Microbiology and Immunology, University of Alberta, Edmonton, Canada; 8 School of Public Health, University of Alberta, Edmonton, Canada; 9 Stollery Science Lab, Edmonton, Canada; 10 Women and Children’s Health Research Institute, Edmonton, Canada

**Keywords:** Solar oxygen, Pediatric pneumonia, Hypoxemia

## Abstract

**Background:**

Child mortality due to pneumonia is a major global health problem and is associated with hypoxemia. Access to safe and continuous oxygen therapy can reduce mortality; however, low-income countries may lack the necessary resources for oxygen delivery. We have previously demonstrated proof-of-concept that solar-powered oxygen (SPO_2_) delivery can reliably provide medical oxygen remote settings with minimal access to electricity. This study aims to demonstrate the efficacy of SPO_2_ in children hospitalized with acute hypoxemic respiratory illness across Uganda.

**Methods:**

*Objectives*: Demonstrate efficacy of SPO_2_ in children hospitalized with acute hypoxemic respiratory illness. *Study design:* Multi-center, stepped-wedge cluster-randomized trial. *Setting:* Twenty health facilities across Uganda, a low-income, high-burden country for pediatric pneumonia. *Site selection:* Facilities with pediatric inpatient services lacking consistent O_2_ supply on pediatric wards. *Participants:* Children aged < 5 years hospitalized with hypoxemia (saturation < 92%) warranting hospital admission based on clinical judgement. *Randomization methods:* Random installation order generated *a priori* with allocation concealment. *Study procedure:* Patients receive standard of care within pediatric wards with or without SPO_2_ system installed. *Outcome measures:* Primary: 48-h mortality. Secondary: safety, efficacy, SPO_2_ system functionality, operating costs, nursing knowledge, skills, and retention for oxygen administration. *Statistical analysis of primary outcome:* Linear mixed effects logistic regression model with 48-h mortality (dependent variable) as a function of SPO_2_ treatment (before versus after installation), while adjusting for confounding effects of calendar time (fixed effect) and site (random effect). *Sample size:* 2400 patients across 20 health facilities, predicted to provide 80% power to detect a 35% reduction in mortality after introduction of SPO_2_, based on a computer simulation of > 5000 trials.

**Discussion:**

Overall, our study aims to demonstrate mortality benefit of SPO_2_ relative to standard (unreliable) oxygen delivery. The innovative trial design (stepped-wedge, cluster-randomized) is supported by a computer simulation. Capacity building for nursing care and oxygen therapy is a non-scientific objective of the study. If successful, SPO_2_ could be scaled across a variety of resource-constrained remote or rural settings in sub-Saharan Africa and beyond.

**Trial registration:**

Clinicaltrials.gov, NCT03851783. Registered on 22 February 2019.

## Background

Globally, approximately 7.7 million children per year die before the age of five years. Infectious diseases account for a large proportion of these deaths, with pneumonia being the leading cause of mortality (2.1 million deaths/year) [1]. Most deaths occur in resource-constrained settings in Asia and Africa [2]. These countries report 2–10 times more children with pneumonia than industrialized countries [3]. In Uganda alone, child mortality is estimated to be 145,000 deaths per year [4]. Bacterial pneumonia, tuberculosis, sepsis, and severe malaria are common infectious etiologies, all of which lead to respiratory distress as a final common pathway. Oxygen (O_2_) therapy is essential to support life in these patients.

Large gaps remain in the clinical management of children presenting to African hospitals with respiratory distress, including essential supportive therapies such as supplemental O_2_. Despite being listed on the World Health Organization’s (WHO) list of essential medicines [5], O_2_ may not be available in hospitals and health centers in low- and middle-income countries (LMICs) because of cost and/or logistical challenges [6, 7]. Methods currently used in low-resource settings include compressed O_2_ cylinders and grid-powered O_2_ concentrators [8, 9]. Cylinders require supply chains linking O_2_ production plants to hospitals which may be compromised by poor road conditions, costs of transportation, and unstable supply chain management [8, 9]. O_2_ losses from cylinders can also vary greatly due to leakage [10, 11]. O_2_ concentrators, while shown to be more cost-effective and user-friendly than cylinders, depend upon a reliable and uninterrupted supply of electricity which is often unavailable in resource-constrained settings [8, 10]. A previous systematic review showed that 26% of health facilities in sub-Saharan Africa reported no access to electricity while only 28% of centers reported reliable access [12].

In resource-constrained settings, O_2_ delivery systems can lead to measurable improvements in survival from childhood pneumonia. A multi-hospital effectiveness study in Papua New Guinea demonstrated a reduction in mortality from childhood pneumonia from 5.0% to 3.2% (35% reduction in mortality) after implementation of an enhanced O_2_ delivery system [13]. We have previously described and implemented a novel strategy for O_2_ delivery that could be implemented in remote locations with minimal access to an electrical power supply: solar-powered oxygen (SPO_2_) delivery [14, 15].

SPO_2_ is an effective solution for supplemental O_2_ in low-resource settings [14–16]. Our systems have been described in detail previously and implemented at two hospitals in Uganda [14–16]. In brief, photovoltaic cells installed on the roofs of hospitals collect solar energy, which is stored as electricity in a battery bank, then used to power an O_2_ concentrator for production of medical grade O_2_. We previously demonstrated the feasibility, safety, and efficacy of SPO_2_ through a proof-of-concept study and a randomized controlled trial (RCT), showing clinical non-inferiority compared to cylinder O_2_ [14–16]. We enrolled 130 children with hypoxemia admitted to two Ugandan hospitals and showed that the length of stay was not prolonged in patients randomized to SPO_2_, relative to children randomized to cylinder O_2_ [15]. Further, we did not detect statistically significant differences in mortality between SPO_2_ and cylinder O_2_. Given its efficacy, before the widespread implementation of SPO_2_ across Africa and Asia, an evaluation of its impact on decreasing the mortality of children admitted with acute hypoxemic respiratory illness is required.

## Methods and design

### Objectives

This study aims to demonstrate the efficacy of SPO_2_ in children hospitalized with acute hypoxemic respiratory illness in Uganda. The study is a multicenter prospective evaluation of SPO_2_, using a stepped-wedge cluster-randomized design.

Our primary aim is to compare the 48-h mortality among children aged < 5 years admitted with hypoxemia before and after the implementation of SPO_2_ delivery at 20 hospitals in Uganda, adjusting for confounding effects of calendar time and site. As secondary aims, we will compare safety and efficacy outcomes among participants, monitor SPO_2_ system functionality and operational costs, and build capacity among nurses to deliver O_2_. The working hypothesis is that SPO_2_ can decrease the mortality of children admitted with hypoxemia Additional files 1 and 2.

### Study design

**Fig. 1:**
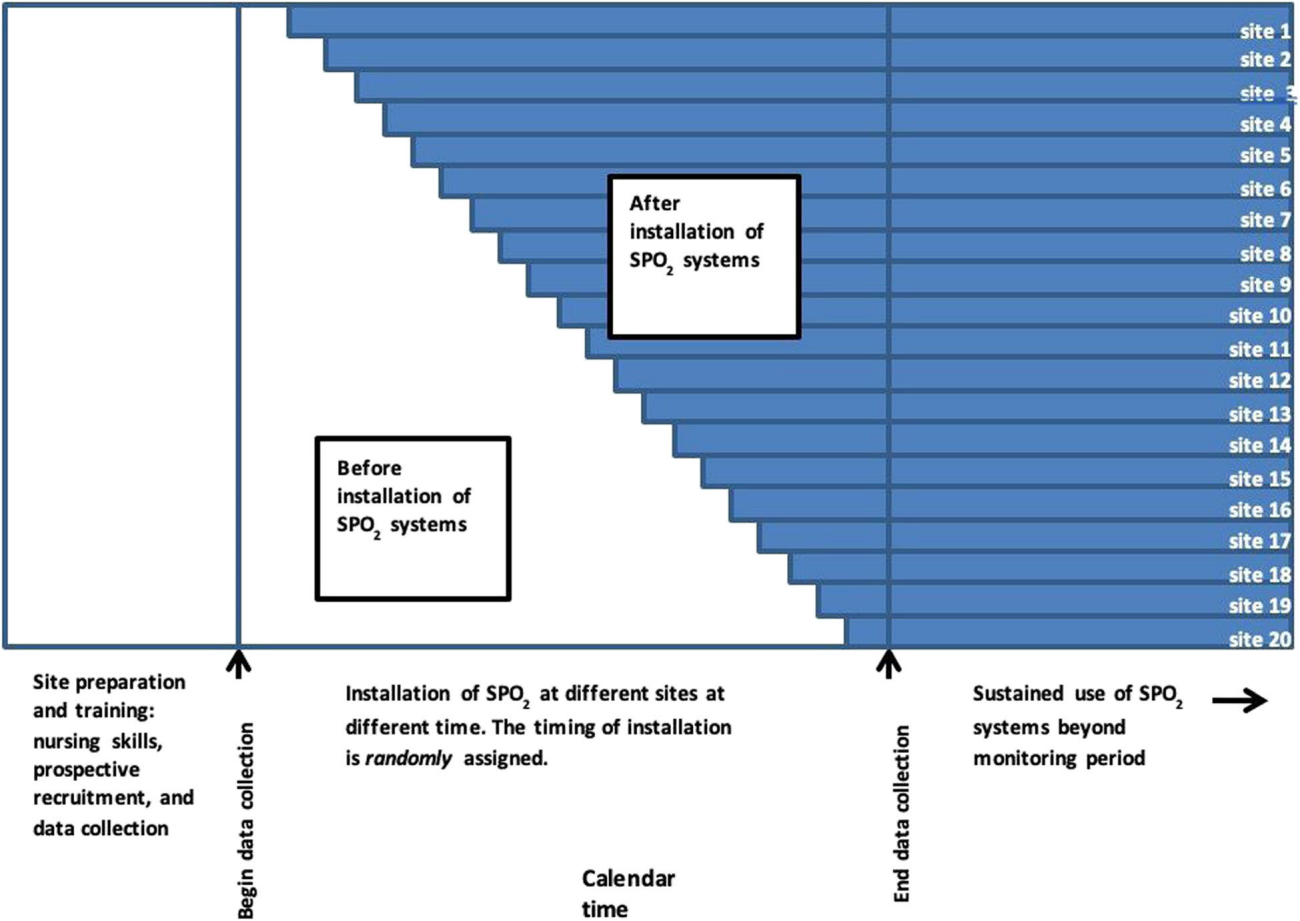
Timeline for cluster-randomized stepped-wedge controlled clinical trial. The trial will involve 20 health facilities and 20 steps. Each step involves enrollment of patients at all sites for one month, with one new site installing SPO_2_ per month. Equipment installed at the health facilities will provide improved access to medical oxygen beyond the life of the study

The study will be a stepped-wedge cluster RCT. This trial will not be blinded. Clusters (health facilities) will be randomly allocated to the timing of implementation of the SPO_2_ system. The installation of SPO_2_ will not be simultaneous but will proceed in accordance with the stepped-wedge design (Fig. 1). All health facilities will have a run-in period during which nurses will be trained in pulse oximetry, O_2_ management, and the study protocol. Data collection (prospective enrolment and accurate electronic record-keeping) will be in place by the end of the run-in period. SPO_2_ will be introduced one site at a time, on a monthly basis, until all 20 sites have SPO_2_ installed. The timing of installation of SPO_2_ will be random, with allocation concealment. Analysis will compare mortality before and after implementation of SPO_2_, using linear mixed effects (LME) logistic regression, adjusting for the confounding effects of time (fixed effect) and site (random effect) [17]. Data collection will continue until the trial’s termination, considered to be one month after the installation of SPO_2_ in the 20th site.

### Setting

Uganda counts approximately 145,000 child deaths annually, of which 16% are attributed to pneumonia [4]. Uganda’s public healthcare system consists of health centers (levels II–IV), general/provincial hospitals, regional referral hospitals, and the National Referral Hospital. The majority of deaths occur in a limited number of general hospitals and level IV health centers where at least half have poor access to a stable electrical supply or O2 cylinders [18]. These represent ideal sites for early implementation of SPO_2_ because of a context-specific need for an O_2_ delivery system that does not rely on electrical power or cylinder distribution. We will enroll 20 sites in our cluster-randomized trial.

### Site selection

Health facilities were evaluated and selected based on the following criteria. Sites were included if they: (1) had pediatric inpatient services; (2) lacked consistent O_2_ supply on pediatric wards; and (3) had adequate space and willingness to install solar panels, a battery bank, and O_2_ concentrator on the hospital premises (i.e. on pediatric ward). Sites were excluded from the study if: (1) had no inpatient services for children; (2) had pre-existing, functional, and consistent O_2_ delivery systems (cylinder or concentrator); (3) did not have space or were unwilling to install solar panels, a battery bank, and O_2_ concentrator.

**Fig. 2:**
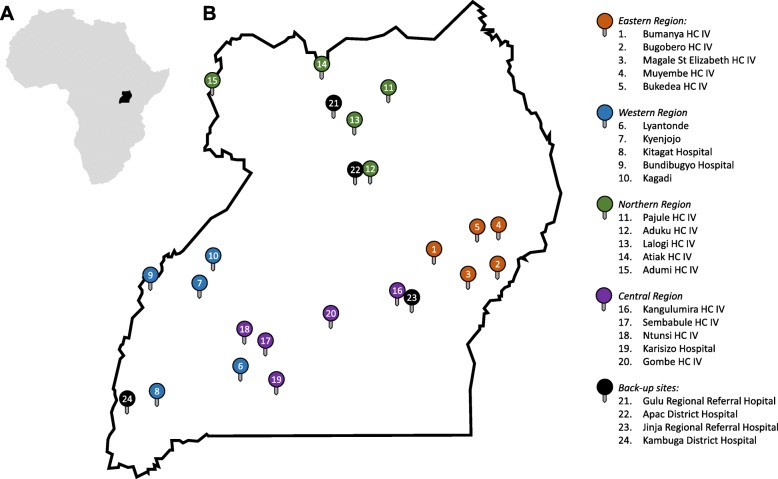
Sites for implementation and evaluation of solar powered O_2_ (SPO_2_) delivery. **a** Map of Africa showing Uganda (*black*). **b** *Map* of Uganda showing SPO_2_ sites. Between four and six health facilities (District Hospitals or Level 4 Health Centers, HC IV) were chosen from each region (Central, Western, Eastern, and Northern) using the following criteria: facility has a pediatric inpatient ward; consistent O_2_ supply on pediatric ward is lacking; adequate space and willingness to install SPO_2_ system on pediatric ward. Sites are geographically distributed across the country and are likely representative of health facilities in sub-Saharan Africa where SPO_2_ would be cost-effective

We conducted site visits to 44 health facilities across the country and interviewed key informants at each site. Based on site selection criteria, five sites were chosen from each of Uganda’s four geographic regions, for a total of 20 sites (Fig. 2). Additional “back-up” sites have also been identified, in case of difficulties with site re-engagement, site/personnel recruitment, or installation at one or more of the primary sites. Characteristics of the 20 chosen sites were as follows. While cylinder oxygen was available at 9/20 (45%) of sites, dedicated cylinders were not available on the pediatric wards of any health facility. Oxygen concentrators were present at 18/20 (90%) of sites, with only 4/18 (22%) of sites with dedicated concentrators available on pediatric wards. Concentrators were shared across wards in 11/18 (61%) sites. At the time of the interview, 11/18 (61%) concentrators were operational, and only 4/18 (22%) produced oxygen with ≥ 80% purity. Power outages were reported as frequent in 17/20 (85%) of sites. While 19/20 (95%) of sites had generators available, none turned them on when children needed oxygen. Because electricity is necessary to run oxygen concentrators, this suggested that access to a reliable power source is a major obstacle in delivering oxygen therapy. Overall, only 3/20 (15%) of sites had oxygen available for pediatric use.

Qualified site champions (local nursing leads) were recruited for each site to support SPO_2_ implementation.

### Participants

We will include patients presenting to the selected sites meeting the following inclusion criteria: (1) age < 5 years; (2) hypoxemia (SPO_2_ < 92%) based on non-invasive pulse oximetry taken within 24 h of admission, on room air; and (3) warrant hospital admission based on clinical judgement. Patients will be excluded from the study if: (1) measured SPO_2_ ≥ 92%; (2) they can be managed as an outpatient; (3) parents or guardians unwilling to provide written informed consent to participate in study; (4) known cyanotic cardiac condition; and (5) diagnosis of hypoxic ischemic encephalopathy in a neonate.

Screening procedures will be as follows: children aged < 5 years presenting to the participating facilities will be screened for hypoxemia using a portable pulse oximeter if they have cough and/or difficulty breathing. Past medical history will be reviewed with the parent or guardian for known cyanotic cardiac condition and/or hypoxic ischemic encephalopathy. If the patient meets eligibility criteria, the parent or guardian will be approached for informed consent.

### Randomization method

The random order of site installation will be generated before the trial. Site names will be written on paper and placed in sequentially numbered sealed opaque envelopes. Once a month, at the time of site selection for the next SPO_2_ installation, the next envelope will be opened to reveal the site. The envelope will be signed and dated, and all envelopes and records will be kept for quality monitoring purposes.

Randomization will be in blocks of four. Each block will include one site from each of four geographic regions of the country (Northern, Eastern, Western, and Central Regions). The order of the four regions within a block will be random; the selection of a site within the region will be randomly sampled without replacement. Thus, after each sequential block of four, the sites will be balanced by region.

### Study procedures

For eligible participants, the parent or legal guardian will be approached for consent to participate in the study (Additional file 3). If granted, the patient will be admitted to the pediatric ward, where there will be a SPO_2_ system installed or not, according to random timing of installation at each site. All patients will receive standard care for their underlying disease, including antibiotics for pneumonia, intravenous fluids as necessary, blood transfusion as necessary, antipyretics, and any other medical therapy required. The study will ensure there are no stock-outs of essential medications or equipment. Basic demographic and clinical data will be collected from the case admission record, and patients will be followed during their hospital admission. The primary outcome, death at 48 h after admission, will be recorded. Other outcomes could include death after 48 h, discharge, and transfer to another facility. Death at 48 h (versus other) will later be analyzed as a binary variable. Patients discharged or transferred to another facility will be followed up by telephone at 48 h after admission to assess status.

**Fig. 3:**
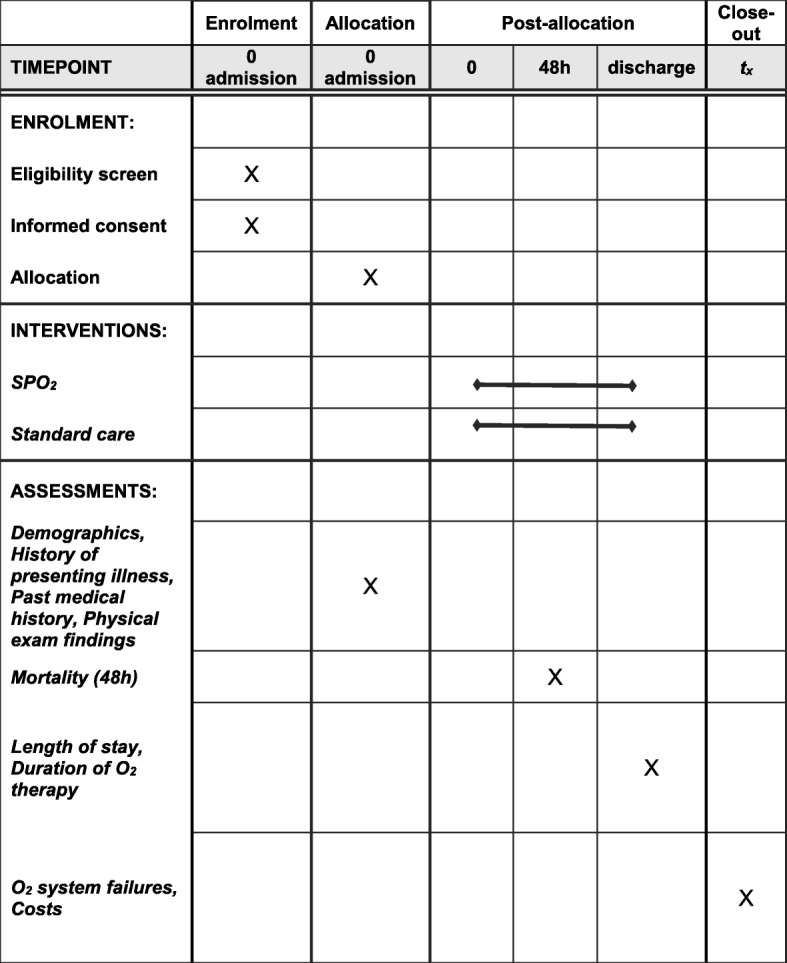
Participant schedule in accordance with SPIRIT 2013 guidelines. The *flow diagram* identifies the time points during the study including Enrolment (admission), Allocation (admission), post-allocation (0, 48 h, discharge), and close-out (end of study). Relevant actions of enrolment, interventions, and assessments performed at the respective time points are marked with an X on the diagram

During hospitalization, secondary outcomes will be recorded, including vital signs at admission and daily thereafter until discharge. Oxygen saturations will be monitored on a 4-hourly basis, during oxygen administration and after. Oxygen utilization (flow rate, duration) will be recorded throughout hospitalization. The need for oxygen therapy will be assessed daily, using standardized criteria for weaning oxygen. The length of stay among survivors will be recorded, as well as the final patient disposition (discharged without disability, discharged with disability, transferred to another facility, or death). The participant flow is illustrated in Fig. 3.

### Outcome measures

Our study’s primary endpoint is mortality at 48 h after admission. We will also evaluate several secondary outcome measures, including in-hospital mortality (time to death), length of hospital stay, duration of supplemental O_2_ therapy (time to wean O_2_), O_2_ delivery system failure(s), cost (installation, servicing, and maintenance), baseline and retained nursing skills in managing oxygen therapy, etiology (based on polymerase chain reaction of dried blood sample and nasopharyngeal swabs), as well as diagnostic and prognostic host biomarkers (Additional file 4).

### Data monitoring

Data will be routinely monitored on each site by a dedicated data collection officer or study nurse, who will be responsible for recording patient information, follow-up, and uploading data to trial databases through KoBoCollect on tablet PCs. During entry, any missing data will be noted on an Errors/Omission log which will be checked regularly by the study team to fill in the missing data. Once uploaded, study data will be securely stored in a locked room and on a password-protected device, accessible only to study personnel. The study monitor or other authorized representatives may inspect all documents and records required to be maintained by the principal investigator; the study site will permit access to such records.

Adverse events (AEs) and unintended effects of SPO_2_ will be monitored, reported, and collected by clinical staff, and will be managed according to national and local standard of care. AEs will be monitored continuously during the study by a dedicated nursing staff. A running log of AEs will be kept and reviewed periodically to allow the study team to assess if any patterns of AEs emerge in real time. All study deaths will require completion of a serious AE form, which will be made available to ethics boards and regulatory authorities within seven days of the event.

No interim data analysis or trial stopping rules are planned for this trial. Oxygen is known to be an essential and life-saving therapy and the efficacy of the SPO_2_ system has been shown to be non-inferior compared to conventional oxygen therapy in the Ugandan context [15]. The stepped-wedge design and commitment to participating sites demands that SPO2 be installed at all sites, such that trial discontinuation at midpoint is not feasible. Because we do not plan to halt the trial before enrolling the planned number of clusters, we have not planned for a Data Monitoring Committee (DMC).

### Statistical considerations

In our trial, we have a cluster-randomized design. The primary dependent variable is the 48-h mortality. The independent (predictor) variable of interest is the exposed/unexposed status of an individual patient (fixed effect), which will depend on whether the patient presents to a participating site before or after installation of SPO_2_. Furthermore, we have covariates which will be modeled as both fixed and random effects. We will use a LME logistic regression model to examine the effect of SPO_2_ on mortality while adjusting for changes in mortality over time (fixed effect) and variability in mortality between sites (random effect). We will use *R* [19] and *lme4* [20] to model the binary outcome (survival) as a function of SPO_2_ treatment, calendar time, and site. As fixed effects, we will enter SPO_2_ treatment (before or after SPO_2_ installation) and calendar time, without interaction term, into the model. We will model site as a random effect. We will determine the statistical significance of the SPO_2_ treatment effect comparing models with and without the SPO_2_ treatment term. If the model fit is significantly improved with inclusion of the treatment term, at α = 0.05 level of significance, we will conclude that the SPO_2_ treatment effect is statistically significant. We will estimate the 95% confidence interval of the treatment effect (odds ratio) from the coefficient of the LME logistic regression model.

Secondary outcomes will be assessed using descriptive and comparative statistics, as appropriate. Where possible, LME models accounting for the clustered data structure and effects of calendar time will be used to determine differences between SPO_2_-treated and untreated patients, with necessary adjustments being made to allow for multiple comparisons within secondary outcomes.

### Sample size

We will include 20 health facilities and a total of 2400 hospitalized patients. This sample size was calculated using a computer simulation, modeling trial conditions and applying the planned statistical analysis. Although analytic methods for sample size calculation for cluster-randomized stepped-wedge trials have been published [21], these require estimates of nuisance parameters (e.g. intra-cluster correlation coefficient) which were not available in the Ugandan context, such that the validity of this method was unknown.

**Fig. 4:**
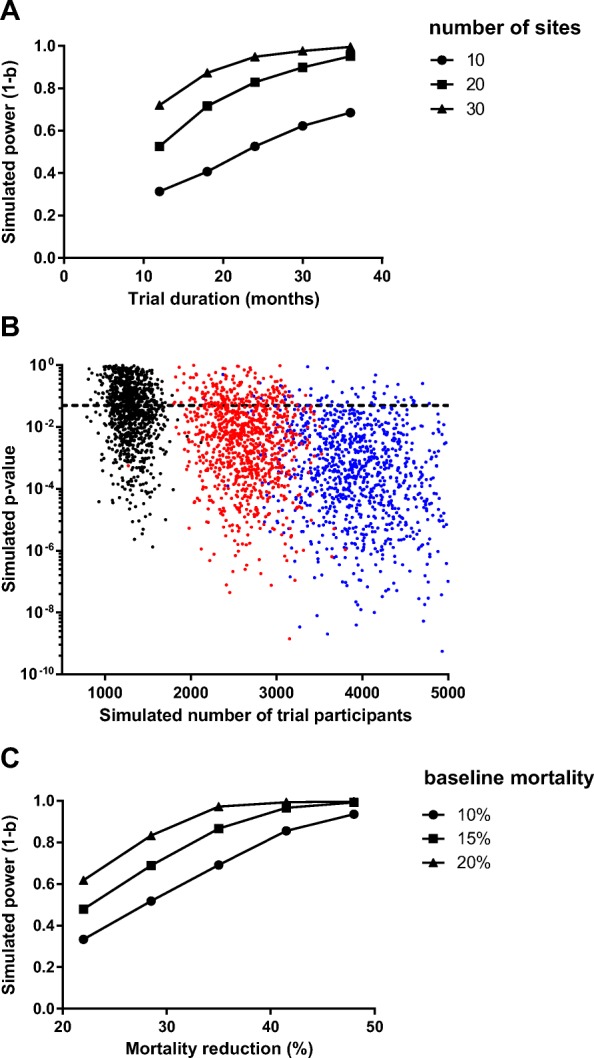
*Computer simulation* for sample size estimation. **a** In the simulation, study power varied with the number of clusters and the duration of enrolment, as expected. Approximately 20 sites enrolling patients over 24 months would provide power of 80%. Each dot represents at least 100 simulated trials. **b** Each dot represents one simulated trial with 20 sites and 20 steps, enrolling patients for two weeks (*black dots*), four weeks (*red dots*), or six weeks (*blue dots*) at each step. The study power is approximated by the proportion of trials appropriately detecting a statistically significant effect of SPO_2_ (*p* < 0.05, *dotted line*). The simulation was repeated 3000 times to generate a plot of *p* value and number of trial participants. For trial simulations with four-week steps (total duration 21 months), the median number of participants enrolled was 2600 (interquartile range 2400–2900) and the statistical power was 82%. **c** In the simulation, study power was also sensitive to variations in the assumptions of baseline mortality and mortality reduction. Our base case (15% baseline mortality, mortality reduction of 35%) was associated with statistical power of 80%. Each dot represents at least 100 simulated trials

For simulation parameters, we used data from the Demographic and Health Information System (DHIS) 2015 for Uganda. This database contains information on the number of patients with clinical pneumonia admitted to each hospital and the number of fatal cases, allowing us to estimate site-specific pneumonia mortality. In a computer simulation, we randomly selected 20 representative sites from the DHIS and used the number of patients admitted with pneumonia at each site to estimate the incidence of hypoxemic patients (13% [22]) that would be enrolled in a hypothetical clinical trial. The monthly number of hypoxemic pneumonia admissions was modeled as a Poisson distribution [23]. Monthly random allocation of SPO_2_ to the 20 sites was simulated, according to the stepped-wedge design. The outcome of each patient at 48 h was modeled as a Bernoulli trial with probability of fatal outcome equal to the site-specific mortality. We used DHIS 2015 data to estimate the baseline mortality assuming a mortality reduction of 35% after installation of SPO_2_ [13]. Having generated simulated trial data, we ran the planned statistical analysis, fitting a LME logistic regression model, as described above. Mortality was modeled as a function of SPO_2_ treatment, with adjustment for covariates of calendar time (fixed effect) and site (random effect), and the *p* value for the treatment effect was calculated. We repeated the simulation > 5000 times and determined the proportion of trials that correctly detected a difference between patients receiving SPO_2_ and those not receiving SPO_2_ (statistical power). Using this simulation strategy, 20 sites enrolling a total of 2400 patients would provide > 80% power to detect a 35% mortality benefit of SPO_2_ at the α = 0.05 level of significance [17, 24]. Data collection could feasibly be completed within 24 months (Fig. 4).

Figure 4a shows the statistical power as a function of the number of sites and the duration of enrolment. Each power estimate was based on at least 100 hypothetical trials in a computer simulation. Approximately 20 sites were required, enrolling patients for two years, to achieve sufficient statistical power. Figure 4b shows individual trial simulations, illustrating increasing power with increasing sample size. Figure 4c shows the sensitivity of the study power to input parameters: baseline mortality and mortality reduction with SPO_2_.

## Discussion

The global burden of pneumonia mortality is concentrated in resource-constrained settings in Africa and Asia [2, 25–27]. Access to O_2_ remains limited and the need for O_2_ has come to the forefront of global health priorities with the WHO recently adding O_2_ to its Model List of Essential Medicines [28]. Several countries, such as Nigeria and Ethiopia, have begun to develop national frameworks for O_2_ scale-up [29, 30]. With increasing recognition of the importance of O_2_ therapy in low-resource settings, novel methods of O_2_ delivery are required. SPO_2_ offers a reliable and sustainable source of O_2_ that could have utility in these low-resource settings. We hypothesize that we will observe a reduction in mortality in children hospitalized with hypoxemia, after installation of SPO_2_, relative to current level of care. We will test this hypothesis in a stepped-wedge cluster-randomized study in Uganda.

SPO_2_ has a number of benefits that lend themselves to further scale-up: reliability in settings with poor access to electricity or O_2_ cylinders [31]; abundance of solar radiation in target LMICs in the tropics [32]; minimal maintenance and training requirements [32]; and cost-effectiveness after a one-time capital investment for installation of equipment. Our group has already demonstrated the feasibility and effectiveness of SPO_2_ at two resource-limited hospitals [14, 15]. The proposed study will demonstrate mortality benefit of SPO_2_ in a country-wide roll-out, providing key data for decision-makers within public health systems with respect to utility (lives saved) and costs of this technology. To our knowledge, only one previous study has quantified the effect size of improved O_2_ delivery on child mortality in a low-resource context [13]. Additional findings from this trial will be of broad interest, including: pneumonia-related mortality at various thresholds of hypoxemia [11]; oxygen utilization at representative health facilities; engineering aspects and costs (installation and maintenance) of SPO_2_ implemented across an entire country; and training strategies in oxygen delivery for frontline nurses.

The choice of study design is motivated by ethical and pragmatic considerations [24, 33]. The cluster-randomized trial design is modern, innovative, and particularly suited to investigations of community level public health interventions that have been proven effective in individual level trials (i.e. “phase IV” effectiveness trials) [17, 24]. Staged roll-out of O_2_ delivery avoids the ethical concern of randomizing individuals to a “no O_2_” arm. All facilities will transition from control to intervention groups [24, 34], providing permanent access to previously unavailable O_2_ therapy by the end of the trial. The staged approach will ease logistical constraints otherwise associated with implementing interventions all together at the same time [24]. With respect to scientific validity, our statistical plan (LME logistic regression model) will adjust for variability in mortality between different sites (random effect) and temporal trends in mortality (fixed effect). Some risks associated with this design were highlighted in a recent systematic review of 46 individual stepped-wedge studies: clusters dropped after randomization or after data collection had started (six studies); delay in implementation of interventions (four studies); and clusters not receiving the intervention at all (five studies) [33]. To mitigate this risk, we have selected two back-up sites that may be recruited in case of cluster dropout. Although this mitigating strategy compromises the stepped-wedge design, it may be necessary to achieve the desired sample size. In case of cluster dropout and addition of new clusters, the statistical plan will include sensitivity analyses including sites planned *a priori* and a pragmatic analysis of all sites ultimately included.

Our study sample size was calculated via a computer simulation using real-world parameters using the Uganda DHIS [22, 35], data from previous large-scale studies [31], and systematic reviews [22]. We applied our planned primary analysis to the simulated data for > 5000 trials to estimate a required sample size of 2400 patients from 20 sites, with 20 steps, recruited over two years to detect a mortality benefit estimated at 35% with 80% power (α = 0.05). Other published stepped-wedge trials in a recent systematic review were of comparable size: median number of participants of 1720 (range 16–292,000), 2–190 clusters, 2–15 steps, over a median period of 20 months (range 9 days to 4 years). Our approach, using computer simulation, has been recommended by previous authors, who noted advantages of simulation-based methods to overcome some of the limitations of analytical formulae and in dealing with the specific features of the study [36].

In addition to our scientific objectives, this study aims to build capacity among frontline nurses for monitoring and delivery of supplemental O_2_ therapy, including pulse oximetry. A study conducted to assess pulse oximetry knowledge and training needs in ward nurses and doctors concluded that only 16% of participants had received any formal training for pulse oximetry and 65% of the participants expressed the need for more training [37]. In another report from Uganda, most nurses were comfortable with the use of oxygen concentrators but were not familiar with pulse oximetry [6]. To address this gap, recognizing context-specific challenges of implementing pulse oximetry [38], SPO_2_ study will train nursing staff in pulse oximetry and safe delivery of O_2_. With enhanced knowledge, skills, and availability of equipment (pulse oximeters and SPO_2_ equipment), our study will improve the quality of care provided to hypoxemic patients during the study and after the study has ended.

Our study has several limitations. The multi-center stepped-wedge design involving health facilities within the public sector, is vulnerable to some external challenges that may not be directly influenced by the researchers (e.g. change of hospital staff or withdrawal of clusters from the study) [33, 39]. The health facilities participating in this study will be in rural or remote communities in Uganda, such that generalizability of our findings to urban or resource-intensive facilities will be limited. The etiology of hypoxemic illness will not be precisely identified. Radiographic confirmation of pneumonia and microbiological confirmation of the infectious etiology would be desirable, but the requisite radiology and lab services are not available in most centers where SPO_2_ will be installed. Additional laboratory testing such as blood culture, sputum culture, viral studies, and a panel of infectious diseases serology would be necessary to definitively diagnose conditions such as pneumococcal pneumonia and viral respiratory tract infections. However, resource limitations preclude an exhaustive battery of diagnostic tests in our setting. Although precise microbiological etiology may not be known, mortality (primary outcome) as well as length of stay (secondary outcome) are clinically meaningful outcomes that can be assessed unambiguously in the context of our study. This study will allow us to demonstrate the mortality benefit of SPO_2_ for treatment of hypoxic respiratory illness, irrespective of etiology, and is similar to the diagnostic capacity of other health facilities where SPO_2_ would potentially be implemented.

Given the magnitude of pediatric pneumonia deaths, estimated at 900,000 per year [40], a life-saving, cost-effective intervention such as SPO_2_ could represent an important tool for the improvement of global child survival. Demonstrating a mortality benefit of SPO_2_ will provide strong supportive evidence for the system and could catalyze the widespread implementation of SPO_2_ in resource-limited settings across Africa and Asia.

## Trial status

Currently recruiting.

Protocol version number 2.2, dated 21 April 2019

Date that recruitment began: 1 July 2019

Anticipated date that recruitment will be completed: 1 July 2021

## Supplementary information

**Additional file 1.** World Health Organization Trial Registration Data Set.

**Additional file 2.** Spirit Checklist.

**Additional file 3.** Consent Form.

**Additional file 4.** Biological Specimens & Ancillary Studies.

## Data Availability

De-identified individual clinical trial participant level data will be shared upon reasonable request submitted to the corresponding author. Findings will be disseminated through presentations at international conferences and will be published in open-access peer-reviewed journals for broad readership and to ensure that the data are available to health workers in low-and middle-income countries. At the conclusion of the study, we will hold a “town hall” meeting at participating health facilities, where local and global study data will be shared with the community members, with opportunities to ask questions. Code (R program) for the computer simulation is available from the corresponding author on reasonable request.

## Abbreviations

DHIS: Demographics and Health Information Systems
LME: Linear mixed effects
LMICs: Low- and middle-income countries
O_2_: Oxygen
SPO_2_: Solar-powered oxygen
