# Supplementary material for: Solar-powered oxygen delivery for the treatment of children with hypoxemia: protocol for a cluster-randomized stepped-wedge controlled trial in Uganda
Source: Trials. 2019 Dec 5;20:679. doi: 10.1186/s13063-019-3752-2 (PMC6896330; doi:10.1186/s13063-019-3752-2)
Supplement: Supplementary file 1 — Additional file 1. World Health Organization Trial Registration Data Set. [file 13063_2019_3752_MOESM1_ESM.pdf]

WORLD HEALTH ORGANIZATION TRIAL REGISTRATION DATA SET

DATA CATEGORY	INFORMATION
**Primary registry and trial identifying number**	ClinicalTrials.gov<br>NCT03851783
**Date of registration in primary registry**	February 22, 2019
**Secondary identifying numbers**	Grand Challenges Canada: 1909-27795<br>Women and Children's Health Research Institute: WCHSSLDRP 2371
**Source(s) of monetary or material support**	Grand Challenges Canada<br>Women and Children's Health Research Institute
**Primary sponsor**	Dr. Michael Hawkes (Investigator Initiated Study)
**Secondary sponsor(s)**	– University of Alberta<br>– Makerere University<br>– Ministry of Health Uganda
**Contact for public queries**	Dr. Michael Hawkes; Telephone (+17802485540); Email (mthawkes@ualberta.ca), 3-588 Edmonton Clinic Health Academy, Faculty of Medicine, University of Alberta, Edmonton, Alberta, Canada
**Contact for scientific queries**	Dr. Michael Hawkes; Telephone (+17802485540); Email (mthawkes@ualberta.ca), 3-588 Edmonton Clinic Health Academy, Faculty of Medicine, University of Alberta, Edmonton, Alberta, Canada
**Public title**	Effect of Solar Powered Oxygen Delivery on Childhood Mortality in Uganda
**Scientific title**	Effect of Solar Powered Oxygen Delivery on Childhood Mortality in Uganda: a cluster randomized trial
**Countries of recruitment**	Uganda
**Health condition(s) or problem(s) studied**	Pneumonia, hypoxemia
**Intervention(s)**	*Intervention:* Solar Powered Oxygen - Our system provides medical-grade oxygen to pediatric patients with hypoxemia, utilizing solar energy to power an oxygen concentrator to deliver therapeutic oxygen. An array of photovoltaic cells regulated by a charge controller, generates electricity, and charges a battery bank. A DC→AC inverter then provides power to the oxygen concentrator, which utilizes

DATA CATEGORY	INFORMATION
	pressure swing absorption technology to concentrate medical-grade oxygen from ambient air.<br><br>*Control:* Selected sites yet to implement the solar powered oxygen delivery system.
**Key inclusion and exclusion criteria**	*Inclusion Criteria (Hospital – Site Selection):*<br>– Pediatric inpatient services<br>– Lack of consistent oxygen supply on pediatric ward<br>– Adequate space and willingness to install solar panels, battery bank, and concentrator on the hospital premises (pediatric ward).<br><br>*Exclusion Criteria (Hospital – Site Selection):*<br>– No inpatient services for children<br>– Pre-existing, functional, consistent oxygen delivery system (cylinders or concentrator)<br>– No space or unwilling to install solar panels, battery bank, and concentrator<br><br>*Inclusion Criteria (Patients):*<br>– Age < 5 years<br>– Hypoxemia (SpO2 < 92%) based on non-invasive pulse oximetry<br>– Hospital admission warranted based on clinical judgement<br><br>*Exclusion Criteria (Patients):*<br>– SpO2 ≥ 92%<br>– Outpatient management<br>– Denial of consent to participate in study<br>– Known cyanotic cardiac condition<br>– Neonatal diagnosis of hypoxic ischemic encephalopathy
**Study type**	*Type*: Interventional<br>*Allocation*: Cluster Randomized<br>*Intervention Model*: Stepped Wedge<br>*Masking*: None<br>*Primary purpose*: Prevention
**Date of first enrolment**	June 1, 2019
**Target sample size**	*Health Facility Sites:* 20<br><br>*Patient Participants:* 2,400
**Recruitment status**	Pending
**Primary outcome(s)**	Mortality at 48 hours after admission
**Key secondary outcomes**	In-hospital mortality, time of death

DATA CATEGORY	INFORMATION
	Length of hospital stay<br>Duration of supplemental oxygen therapy (time to wean O_2)<br>Oxygen delivery system failure<br>Costs (installation, servicing, maintenance)<br>Nursing skills, baseline and skills retention
**Ethics Review**	Institutional Review Board of the University of Alberta: Approve – February 25, 2019; Telephone (+17804920459); Email (reoffice@ualberta.ca)<br>Biomedical Sciences Research and Ethics Committee of Makerere University: Approved – March 5, 2019; Telephone (+256 75 25 75 050); Email (irbbiomedicalsciences@gmail.com)
**Completion date**	May 31, 2021
**Summary Results**	Not yet enrolled
**IPD Sharing Statement**	De-identified individual clinical trial participant level data (IPD) will be shared upon reasonable request submitted to the corresponding author.<br>Findings will be disseminated through presentations at international conferences, and will be published in open access peer-reviewed journals for broad readership and to ensure that the data are available to health workers in low-middle income countries.<br>At the conclusion of the study, we will hold a “town hall” meeting at participating health facilities, where local and global study data will be shared with the community members, with opportunities to ask questions.
