# Supplementary material for: Solar-powered oxygen delivery for the treatment of children with hypoxemia: protocol for a cluster-randomized stepped-wedge controlled trial in Uganda
Source: Trials. 2019 Dec 5;20:679. doi: 10.1186/s13063-019-3752-2 (PMC6896330; doi:10.1186/s13063-019-3752-2)
Supplement: Supplementary file 3 — Additional file 3. Consent Form. [file 13063_2019_3752_MOESM3_ESM.pdf]

Title of Study:
Effect of Solar Powered Oxygen Delivery on Childhood Mortality in Uganda: a cluster-randomized trial

Consent Form

Date	☐☐/☐☐/☐☐☐	Study ID	

A. Investigators:

This study is being conducted by **Dr. Robert Opoka** from Mulago hospital (Tel no: 0772205329) **Dr. Sophie Namasopo from Kabale Hospital (Tel no: 0772470297)** and **Dr. Michael Hawkes (Tel no: +1-780-248-5540)** from Canada in collaboration with the Ministry of Health of Uganda. We plan to enroll a total of 2400 children in the study across 20 hospitals in Uganda. All children in the study will in addition receive the standard treatment in hospital for their disease.

B. Background and rationale for the study:

Previously, we determined that a new way of delivering oxygen, using solar power, is as effective for giving oxygen to patients as standard oxygen tanks. We are now expanding solar oxygen across Uganda, and we are tracking patients to measure the benefit of bringing solar oxygen to the hospital in terms of lives saved.

C. Description of sponsors of the research project and the organizational affiliation of the researchers:

The study is sponsored by Grand Challenges Canada and Women and Children's Health Research Institute (WCHRI).

D. Study Purpose:

The purpose of this study is to determine whether introducing solar powered oxygen delivery reduces deaths among children with low blood oxygen.

E. The estimated duration the research participant will take to in the research project:

Your child will be in the study until discharged from the hospital (usually about 2-5 days).

F. Study Procedures:

While in hospital, the doctors will treat your child according to standard Ugandan Ministry of Health guidelines. This may include giving oxygen, if available, by nasal cannula or by mask. The hospital where your child is being admitted is one of 20 sites where solar powered oxygen will be installed over a 2-year period; the timing

of installation is randomly assigned between the 20 sites over the 2-year period. We are monitoring children with low blood oxygen during this 2-year period to see the outcome of their illness, before and after installation of solar oxygen.

If you agree to be in the study, we will collect medical information about your child over their stay in hospital including vital signs, how much oxygen they are using, and how long they stay in hospital. We may also do a chest x-ray, nose swab and blood samples for the study, if resources are available: The blood will be removed by venipuncture into 2.0mL pediatric Vacutainer® tubes. The volume of blood drawn will not exceed 2.0mL (half a teaspoon).

We will follow your child in hospital to record the final outcome as well as the length of hospital stay.

G. Who will participate in the study:

Your child is invited to participate in this study because your child is being admitted to hospital and needs oxygen. This study requires 2400 children who are below 5 years and need oxygen.

H. Risks of Study Participation

There are few risks to your child from being in this study. Your child will not receive any treatment for this study other than standard care, including oxygen, if available.

I. Benefits of Study Participation

There is no direct benefit of participation in this study to your child. However, children hospitalized with low oxygen sometimes have very serious disease that can lead to death or disability in survivors. Your child will be carefully monitored during the study, and we will attempt to ensure that treatment is given according to national guidelines. If this study shows that solar powered oxygen is better than oxygen from tanks, then this treatment could be used in the future to treat children with diseases that require oxygen, for example pneumonia, where the power supply is not reliable.

J. Confidentiality

The records of this study will be kept private. In any publications or presentations, we will not include any information that will make it possible to identify you or your child as a subject. The anonymous record of your child might, however, be reviewed by Makerere University and/or the University of Alberta who have authorized this study. No study information will be recorded in the child’s hospital record. Any study data that is to be transmitted via the Internet will be encrypted with secure passwords known to the sender and recipient(s) only.

K. Alternatives

Even if you do not participate in the research, you will still receive the usual health care at the health care facility.

L. Cost

Any tests and treatment costs not provided by the hospital will be covered by the study

M. Compensation for participation in the study

We will provide UGX20, 000 to compensate for your time at the end of the study.

N. Reimbursement

You will not incur any costs by participating in this study. The study will provide transport in case they are called for a study related procedure

O. Questions about the study

For any study related issues/questions now or later, you are encouraged to contact the researchers conducting this study at 0772996164 (Dr. Robert Opoka).

P. Questions about participant's rights

If you have any questions or concerns regarding the study and would like to talk to someone other than the researcher(s), you are encouraged to contact Dr Mwaka Erisa, chairperson school of Biomedical Sciences Research Ethics Committee, on 0752575050, Acting principal, College of health Sciences, Makerere University, on 0775526810, and provide him with the study title.

Q. Statement of voluntariness

Participation in this study is voluntary. Your decision whether or not to participate in this study will not affect your current or future relations with the hospital, Makerere University, or the University of Alberta or your ability to participate in other research studies. Even if you decide to participate, you are free to withdraw at any time without affecting those relationships. Refusing to participate will not alter your/your child's usual health care or involve any penalty or loss of benefits to which you or your child are otherwise entitled.

R. Dissemination of results

You will get feedback on findings and progress of this study and if there is any new information that affects the study or data that has clinical relevance to your child, this will be made available to you and/or your health care providers.

S. Ethical approval

This study was approved by School of Biomedical Sciences Research Ethics Committee and Uganda National Council of Science and Technology.

STATEMENT OF CONSENT/ASSENT

.... has described to me what is going to be done, the risks, the benefits involved and my rights regarding my child's participation in the study. I understand that my decision to participate in this study will not alter my usual medical care. In the use of this information, my identity will be concealed. I am aware that I may withdraw at any time. I understand that by signing this form, I do not waive any of my legal rights but merely indicate that I have been informed about the research study in which I am voluntarily agreeing to my child to participate in. A copy of this form will be provided to me.

Parent/guardian:

Signature or thumbprint: …………………………………… Date…………………………..

Name (print:) ……………………………………

Witness (if applicable):

Signature: …………………………………… Date…………………………..

Name (print:) ……………………………………

Interviewer/Person obtaining informed consent:

Signature: …………………………………… Date…………………………..

Name (print:) ……………………………………
