# Supplementary material for: Solar-powered oxygen delivery for the treatment of children with hypoxemia: protocol for a cluster-randomized stepped-wedge controlled trial in Uganda
Source: Trials. 2019 Dec 5;20:679. doi: 10.1186/s13063-019-3752-2 (PMC6896330; doi:10.1186/s13063-019-3752-2)
Supplement: Supplementary file 4 — Additional file 4. Biological Specimens & Ancillary Studies. [file 13063_2019_3752_MOESM4_ESM.docx]

**BIOLOGICAL SPECIMENS & ANCILLARY STUDIES**

**BIOLOGICAL SAMPLES**

***Dried Blood Spot****:*

- *Purpose:* Blood samples obtained at admission may be analyzed for evidence of pathogens.
- *Collection:* Samples will be collected on filter paper.
- *Evaluation:* Pathogens present in dried blood spots will be detected by multiplex PCR.
- *Storage:* Room temperature

***Nasopharyngeal (NP) Swab:***

- *Purpose:* NP samples obtained at admission may be analyzed for evidence of bacterial and viral pathogens.
- *Collection:* Samples will be collected by study nurses.
- *Evaluation:* Viral pathogens present in nasopharyngeal swabs will be detected by multiplex PCR.
- *Storage:* Samples will be stored in a refrigerated tank containing liquid nitrogen prior to and during transportation to lab for evaluation or a -80°C freezer.

***Serum/ Plasma:***

- *Purpose:* Blood obtained in the course of admission may be analyzed for evidence of bacterial or viral pathogens and/or biomarkers related to pneumonia.
- *Collection:* Samples will be collected by study nurses. Blood will be removed by venipuncture into 2.0mL pediatric Vacutainer® tubes.
- *Evaluation:* Venous lactate levels, analyzed with Lactate Scout Analyzer. Other biomarkers will be assessed by enzyme-linked immunosorbent assay (ELISA) using commercially available kits.
- *Storage:* Samples will be stored in a refrigerated tank containing liquid nitrogen prior to and during transportation to lab for evaluation or a -80°C freezer.

**ANCILLARY STUDIES:**

Samples collected during the period of the SPO_2_ study may also be utilized for the purpose of additional investigation and study. Studies may include:

- ***Etiology of Pneumonia:*** The causative pathogens responsible for childhood pneumonia in low-income settings often remained unidentified due to poor availability of radiographic and microbiological laboratory services within low-resource setting. Without clearly identified pathogens, clinical management of pediatric patients remains challenging. Biological specimens from the SPO_2_ trial may support the identification of viral, bacterial, or fungal pathogens contributing to the development of childhood pneumonia in rural and low-income settings, enabling improved clinical management.
- ***Biomarkers to predict Mortality:*** Our team has previously identified novel biomarkers for childhood pneumonia, including neutrophil gelatinase-associated lipocalin (NGAL), chitinase-3-like-1 (CHI3L1), lipocalin-2 (LCN2), soluble triggering receptor expressed on myeloid cells-1 (sTREM1), and Angiopoietin-2 (ANG2). These are newly described putative biomarkers for diagnosis and prognosis for pneumonia, the leading cause of mortality of children under 5 globally. Leveraging biological specimens from the SPO_2_ trial, our goal is to validate these plasma biomarkers as predictors of fatal outcome among children with suspected pneumonia.
